# Age Effects in Facial Fracture Trauma: Disparities in Multisystem Injuries in Non-Fall-Related Trauma

**DOI:** 10.7759/cureus.48091

**Published:** 2023-11-01

**Authors:** Joseph Boscia, Heather X Rhodes, Thomas Sanders, Saptarshi Biswas

**Affiliations:** 1 Surgery, University of South Carolina School of Medicine Columbia, Columbia, USA; 2 Trauma, Grand Strand Medical Center, Myrtle Beach, USA; 3 Surgery, Grand Strand Medical Center, Myrtle Beach, USA

**Keywords:** social disparities, elderly falls, facial trauma, age effects, facial fracture

## Abstract

Background and objective

Facial fractures represent a growing concern among an aging population prone to falls. In light of this, this study aimed to investigate differential facial fracture patterns and outcomes based on age effects. Determining the differences between the severity and type of facial fractures in populations of different ages will help guide clinical decision-making when managing patients with facial fractures.

Methods

This was a single-center study involving trauma registry data, from July 1, 2016, to January 31, 2022. The inclusion criteria were based on the International Classification of Diseases (ICD-10) diagnosis of facial fracture. A linear regression was performed to ascertain the effects of predictor variables on the likelihood that a facial fracture trauma patient would experience various age effects on injury location, mortality, and morbidity.

Results

A total of 1575 patients were included in the analysis. A significant regression equation was found (F(47,1476)=42.46, p<0.01), with an R^2^ of 0.57. Older facial fracture trauma patients were more likely to be female (β=3.13, p<0.01) with fractures to their zygoma (β=2.57, p=0.02). Higher Abbreviated Injury Scale (AIS) facial region scores (β=2.21, p=0.03), longer hospital length of stay (β=0.07, p=0.02), and in-hospital mortality (β=10.47, p<0.01) were also associated with older age. Older age was additionally associated with a higher level of several morbidity markers. Younger facial fracture trauma patients were more likely to be African American (β=-5.46, p<0.01) or other, non-Caucasian race (β=-8.66, p<0.01) and to have mandible fracture patterns (β=-3.63, p<0.01). The younger patients were more likely to be fully activated (β=-3.10, p<0.01) with a higher shock index ratio (SIR) (β=-7.36, p<0.01). Injury mechanisms in younger facial fracture patients were more likely to be assault (β=-12.43, p<0.01), four-wheeler/ATV accident (β=-24.80, p<0.01), gunshot (β=-15.18, p<0.01), moped accident (β=-13.50, p<0.01), motorcycle accident (β=-12.31, p<0.01), motor vehicle accident (β=-16.52, p<.01), or pedestrian being struck by a motor vehicle (β=-10.69, p=0.02).

Conclusions

Based on our findings, age effects impact facial fracture patterns and outcomes. Younger patients are more likely to experience multisystem injuries via non-fall trauma. On the other hand, older patients are more likely to experience more severe primary facial injuries. Older patients are also at a higher risk of fall-related trauma. Disparities also exist between genders and races, with male and non-Caucasian patients being at a higher risk of injury from facial fractures at a younger age. With an aging population, the prevalence of falls is likely to increase. Thus, facial fractures represent a growing healthcare burden and warrant future investments related to care and treatment.

## Introduction

In 2019, around 10.7 million new cases of facial fractures were reported worldwide [1]. Facial fractures encompass injury patterns affecting the skull's nasal, zygomatic, maxillary, and mandibular bones. These fractures often require complex care and highly specialized physicians to adequately and appropriately diagnose and treat. Thus, facial fractures are associated with a significant strain on the healthcare system.

In the United States and many other parts of the world, rapidly aging populations are leading to a rise in trauma rates [2]. Healthcare systems worldwide are adapting to properly respond to an increased number of trauma-related cases. Fall-related trauma commonly affects aging populations, with falls being the leading cause of injury among adults aged ≥65 years [3,4]. Furthermore, around 30% of people aged ≥65 years experience a fall at some point in their lifetime [3]. 

Facial fracture incidence is most commonly reported in the following two populations: those aged 5-20 years and those older than 70 years [1]. Additionally, these two populations experience different types of facial fractures [5]. Although facial fractures are well-documented in the United States, more research is required to better understand the populations most at risk for different types of facial fractures. This study aims to investigate differences in facial fracture patterns and outcomes based on age effects.

This article was presented as a meeting abstract at the 2023 Southeastern Surgical Congress on February 14, 2023.

## Materials and methods

Study design

This single-center retrospective cohort study was conducted based on data from July 1, 2016, to January 31, 2022. Trauma registry data were retrospectively reviewed to collect patient demographics (age, gender, race, transferred-in details, types of facial fractures, mode of arrival), injury details [trauma activation, injury severity score (ISS), Abbreviated Injury Scale (AIS), region of the face, mode of injury, Glasgow Coma Scale (GCS) score, shock index ratio (SIR), alcohol level (ETOH), mechanism of injury], morbidity details [length of stay in intensive care unit (ICU), ventilation duration, emergency department (ED) intubation, hospital length of stay, discharge disposition], in-hospital complications, and preexisting comorbidity.

Setting and population

The inclusion criteria included all patients with an International Classification of Diseases (ICD-10) diagnosis code for facial fracture admitted to an American College of Surgeons (ACS) adult level I and pediatric level II trauma center in South Carolina. No criteria for patient exclusions were identified in this study. This trauma center is located on the central east coast of the US and treats a large percentage of rural injuries. The catchment area of this trauma center exceeds 95 miles, extending into the southern regions of North Carolina. The annual volume of activated pediatric/adult trauma patients is estimated at 4000 per year.

Data analysis

Descriptive statistics, including mean (standard deviation), median, and frequencies (percentage), were reported for the entire sample. The predictor variables for the regression model were chosen using a multicollinearity test for tolerance with values above 0.5, which satisfied goodness of fit. All variables selected for the model were independent of one another. For all models, p-values less than 0.05 were considered statistically significant. The data were analyzed using a multivariable linear regression in SPSS Statistics version 28 (IBM Corp., Armonk, NY).

The dependent variable was age. Predictor variables included patient demographics (gender, race, transferred-in details, types of facial fractures, mode of arrival), injury details (trauma activation, ISS, AIS region of the face, mode of injury, GCS score, SIR, alcohol level, mechanism of injury), morbidity details (length of stay in ICU, ventilation days, ED intubation, hospital length of stay, discharge disposition), in-hospital complications, and preexisting comorbidity. This research was determined to be exempt from the HCA Grand Strand Medical Center Institutional Review Board oversight in accordance with current regulations. Investigators were only provided with a limited/de-identified dataset. Injuries, causes, and procedures were coded using ICD-10 codes; the AIS system was used for additional diagnostic and injury severity scoring.

## Results

A total of 1575 patients (mean age: 51 years) were included in the analysis. Five types of facial fracture locations were identified: orbit (36%), zygoma (15%), maxillary (28%), mandible (15%), and other (nasal, teeth, etc., 59%). The majority of the data involved Caucasian (79%) males (67%) who were transported by ground EMS (82%) and partially activated (45%) (Table 1). The majority of the injury patterns pertained to blunt (95%) and fall trauma (41%) with a mean ISS of 12 (SD=10), GCS score of 12 (SD=4), and shock index of 0.64 (SD=0.31) (Tables 1, 2). A significant regression equation was found (F(47,1476)=42.46, p<0.01), with an R^2^ of 0.57.

**Table 1:** Characteristics of all trauma patients diagnosed with a facial fracture (N=1575) HEMS: helicopter emergency medical services; ISS: injury severity score; AIS: Abbreviated Injury Scale; GCS: Glasgow Coma Scale; SIR: shock index ratio; ETOH: alcohol; ATV: all-terrain vehicle; COPD: chronic obstructive pulmonary disease; CPR: cardiopulmonary resuscitation; DVT/PE: deep vein thrombosis/pulmonary embolism

Variables	N	%
Mean age: 51 years		
Gender		
Male	1067	67%
Female	508	32%
Race		
African American	227	14%
Caucasian	1254	79%
Other	67	4%
Transferred in	350	22%
Elapsed time at referral, hours	274	
Types of facial fractures		
Orbit	575	36%
Zygoma	247	15%
Maxillary	450	28%
Mandible	239	15%
Other (nasal, teeth, etc.)	935	59%
Mode of arrival		
Ground	1305	82%
HEMS	141	8%
Private vehicle/walk-in	123	7%
Trauma activation		
Full	612	38%
Partial	721	45%
Consult	192	12%
None	50	3%
ISS	1575	
AIS region face	1571	
GCS	1553	
SIR	1529	
ETOH level	528	33%
Mode of injury		
Asphyxiation	1	0.06%
Assault	205	13%
Bicycle accident	23	1%
Fall	648	41%
Four-wheeler/ATV accident	23	1%
GSW	52	3%
Industrial	4	0.20%
Injury by animal	2	0.10%
Moped accident	46	2%
Motor vehicle-related Injury	4	0.20%
MCC	170	10%
MVC	288	18%
Pedestrian struck by MVC	54	3%
Sports	11	0.60%
Stab	3	0.10%
Other	29	1%
Mechanism of injury		
Blunt	1504	95%
Penetrating	67	4%
ICU length of stay, days	1575	
Ventilator days	1573	
ED intubated	182	11%
Hospital length of stay, days	1575	
Discharge disposition		
Expired	76	4%
Home without care	1023	64%
Home under the care of home health	166	10%
Short-term general hospital	42	2%
Intermediate care facility	7	0.40%
Court/law enforcement	16	1%
Hospice	23	1%
Inpatient rehab	95	6%
Long-term care hospital	30	1%
Skilled nursing facility	53	3%
Psychiatric hospital	1	0.06%
Left against medical advice	28	1%
Another type of institution not defined	14	0.80%
Preexisting comorbidity		
Alcohol use disorder	87	5%
Anticoagulation therapy	173	10%
COPD	64	4%
Congestive heart failure	44	2%
Current smoker	271	17%
Dementia	57	3%
Diabetes mellitus	165	10%
Hypertension	424	26%
Personality disorders	91	5%
Complications		
Cardiac arrest w/CPR	18	1%
DVT/PE	35	2%
Unplanned intubation	13	0.80%
Unplanned admission to ICU	23	1%
Ventilator-associated pneumonia	17	1%

**Table 2:** Descriptive statistics of trauma patients diagnosed with a facial fracture This table shows central tendency and dispersion measures for trauma patients diagnosed with facial fractures; X̄: mean, M: median ISS: injury severity score; AIS: Abbreviated Injury Scale; GCS: Glasgow Coma Scale; SIR: shock index ratio; ETOH: alcohol

Variables	X̄	M	[SD]R	Min	Max
Age, years	51	52	22	0	100
Elapsed time at referral, hours	165	152	91	1	548
ISS	12	9	10	1	75
AIS region face	1.63	2	0.51	1	3
GCS	12	15	4	3	15
SIR	0.64	0.6	0.31	0.02	8.69
ETOH level, mg/dL	169	178	103	0	487
ICU length of stay, days	3.07	0	6.32	0	79
Ventilator days	2	0	7.2	0	137
Hospital length of stay, days	6.36	2	14	1	182

Older facial fracture trauma patients were more likely to be female (β=3.13, p<0.01), with fractures to their zygoma (β=2.57, p=0.02), higher AIS facial region scores (which indicate the severity of the facial fracture itself) (β=2.21, p=0.03), and longer hospital length of stay (β=.07, p=0.02), who experienced in-hospital mortality (β=10.47, p<0.01), with a higher level of morbidity markers, such as a discharge to home healthcare (β=3.45, p<0.01), hospice (β=11.42, p<0.01), inpatient rehab (β=7.65, p<0.01), long-term care hospital (β=8.05, p<0.01), or skilled nursing facility (β=5.65, p=0.01) (Table 3).

**Table 3:** Linear regression analysis of all trauma patients diagnosed with a facial fracture This table shows the correlation between outcome/dependent variables and age as determined by linear regression analysis A significant regression equation was found (F(47,1476)=42.46, p<0.01), with an R^2^ of 0.57 HEMS: helicopter emergency medical services; AIS: Abbreviated Injury Scale; GCS: Glasgow Coma Scale; SIR: shock index ratio; ATV: all-terrain vehicle; COPD: chronic obstructive pulmonary disease; CPR: cardiopulmonary resuscitation; DVT/PE: deep vein thrombosis/pulmonary embolism

Outcome/dependent variable: age							
	Unstandardized coefficients					95% CI for B	
	B	Std. error	Standardized coefficients Beta	t	Sig.	Lower bound	Upper bound
Constant	51.93	2.97		17.44	<0.01	46.09	57.77
Sex	3.13	0.87	0.06	3.57	<0.01	1.41	4.85
Race							
African American	-5.46	1.16	-0.08	-4.67	<0.01	-7.75	-3.17
Caucasian	Baseline						
Other	-8.66	1.95	-0.07	-4.44	<0.01	-12.48	-4.84
Transferred In	-0.16	0.99	-0.00	-0.16	0.87	-2.11	1.79
Types of facial fractures							
Orbit	-0.98	1.05	-0.02	-0.93	0.35	-3.05	1.08
Zygoma	2.57	1.17	0.04	2.19	0.02	0.27	4.88
Maxillary	-0.97	1.02	-0.02	-0.95	0.33	-2.98	1.02
Mandible	-3.63	1.18	-0.05	-3.06	<0.01	-5.96	-1.31
Other (nasal, teeth, etc.)	0.70	0.92	0.01	0.76	0.44	-1.10	2.50
Mode of arrival							
Ground	Baseline						
HEMS	-0.42	1.47	-0.00	-0.28	0.77	-3.32	2.47
Private vehicle/walk-in	-1.96	1.50	-0.02	-1.30	0.19	-4.92	0.99
Trauma activation							
Full	-3.10	1.06	-0.06	-2.91	<0.01	-5.19	-1.01
Partial	Baseline						
AIS region face	2.21	1.00	0.04	2.10	0.03	0.14	4.10
GCS	0.23	0.13	0.04	1.75	0.08	-0.02	0.50
SIR	-7.36	1.33	-0.10	-5.51	<0.01	-9.98	-4.75
Mode of Injury							
Assault	-12.43	1.35	-0.18	-9.19	<0.01	-15.08	-9.78
Bicycle accident	-5.85	3.29	-0.03	-1.77	0.07	-12.32	0.61
Fall	Baseline						
Four-wheeler/ATV	-24.80	3.28	-0.13	-7.54	<0.01	-31.25	-18.35
GSW	-15.18	2.46	-0.12	-6.16	<0.01	-20.01	-10.34
Moped	-13.50	2.41	-0.10	-5.59	<0.01	-18.23	-8.77
MCC	-12.31	1.45	-0.17	-8.48	<0.01	-15.16	-9.46
MVC	-16.52	1.25	-0.28	-13.19	<0.01	-18.98	-14.07
Pedestrian struck by MVC	-10.69	2.26	-0.08	-4.72	<0.01	-15.14	-6.25
Hospital length of stay, days	0.07	0.03	0.04	2.18	0.02	0.00	0.14
Discharge disposition							
Expired	10.47	2.21	0.10	4.73	<0.01	6.13	14.80
Home without care	Baseline						
Home under the care of home health	3.45	1.32	0.04	2.60	<0.01	0.84	6.05
Short-term general hospital	-3.87	2.44	-0.02	-1.58	0.11	-8.65	0.91
Court/law enforcement	1.99	3.86	0.00	0.51	0.60	-5.59	9.58
Hospice	11.42	3.34	0.06	3.41	<0.01	4.86	17.98
Inpatient rehab	7.65	1.73	0.08	4.42	<0.01	4.25	11.04
Long-term care hospital	8.05	3.02	0.04	2.65	<0.01	2.10	13.99
Skilled nursing facility	5.65	2.28	0.04	2.47	0.01	1.16	10.13
Left against medical advice	2.24	2.95	0.01	0.76	0.44	-3.55	8.04
Preexisting comorbidity							
Alcohol use disorder	0.89	1.73	0.00	0.51	0.60	-2.50	4.29
Anticoagulation therapy	10.48	1.34	0.14	7.78	<0.01	7.84	13.12
COPD	7.38	2.02	0.06	3.65	<0.01	3.42	11.35
Congestive heart failure	3.79	2.45	0.02	1.54	0.12	-1.02	8.61
Current smoker	-2.58	1.10	-0.04	-2.34	0.01	-4.74	-0.42
Dementia	11.67	2.16	0.09	5.38	<0.01	7.42	15.91
Diabetes mellitus	2.13	1.35	0.02	1.58	0.11	-0.51	4.78
Hypertension	12.75	1.01	0.25	12.55	<0.01	10.75	14.74
Personality disorders	0.52	1.68	0.00	0.31	0.75	-2.78	3.83
Complications							
Cardiac arrest w/CPR	-5.18	3.85	-0.02	-1.34	0.17	-12.73	2.37
DVT/PE	1.31	3.13	0.00	0.42	0.67	-4.82	7.46
Unplanned intubation	1.90	4.48	0.00	0.42	0.67	-6.88	10.68
Unplanned admission to ICU	4.02	3.51	0.02	1.14	0.25	-2.87	10.92
Ventilator-associated pneumonia	-3.63	4.24	-0.01	-0.85	0.39	-11.95	4.68

Younger facial fracture trauma patients were more likely to be African American (β=-5.46, p<0.01) or other race (β=-8.66, p<0.01) with mandible fracture patterns (β=-3.63, p<0.01) (Table 3). The younger patients were likely fully activated (β=-3.10, p<0.01) with a higher shock index ratio (β=-7.36, p<0.01) (Table 3).

The modes of injury patterns in the younger facial fracture trauma population were likely to be assault (β=-12.43, p<0.01), four-wheeler/ATV accident (β=-24.80, p<0.01), gunshot (β=-15.18, p<0.01), moped accident (β=-13.50, p<0.01), motorcycle accident (β=-12.31, p<0.01), motor vehicle accident (β=-16.52, p<0.01), or pedestrian struck by a motor vehicle (β=-10.69, p=0.02) (Table 3).

Older facial fracture patients were more likely to have a pre-existing condition related to anticoagulation therapy (β=10.48, p<0.01), COPD (β=7.38, p<0.01), dementia (β=11.67, p<0.01) or hypertension (β=12.75, p<0.01) (Table 3). Younger facial fracture trauma patients were more likely to be current smokers (β=-2.58, p=0.01) (Table 3).

## Discussion

Although facial fractures are well-documented, differences between populations based on age have not been investigated until recently. This study aims to provide deeper insights into observed differences in facial fractures between populations based on age. We identify variables that influence the age at which people experience facial fractures. We present several significant findings by examining various demographics, injury mechanisms, and other variables. 

It has been well-reported that older patients are at a greater risk of falls [3,4]. In this study, facial fracture patients experiencing falls were significantly older on average compared to patients experiencing assault, gunshot wounds, any kind of vehicular accident (including motor vehicle, motorcycle, moped, or four-wheeler/ATV accident), or when a pedestrian is struck by a motor vehicle. This supports the finding that older patients are at a greater risk for falls and also demonstrates the relatively high risk of the trauma types in younger facial fracture patients compared to falls. Risk-taking behavior is associated with younger individuals [6]. Thus, risk-taking behavior may account for why younger people are more likely to experience non-fall-related trauma. That the AIS region of the face is significantly higher and the shock index ratio is significantly lower in older patients supports the idea that older patients experience injury mechanisms that primarily affect the face rather than those causing widespread systemic injuries. This claim is supported by the finding that falls often result in facial fractures as the primary injury [7]. SIR decreases as the patient's age goes up. This supports the claim that younger facial fracture patients are more likely to experience multisystem trauma as they present with more systemic injuries than older patients. Patients are more likely to be older when the primary injury or the most severe injury is the facial fracture itself. In contrast, younger facial fracture patients are more likely to require a fully activated trauma response. This corresponds to more complex or severe traumas. This is likely a reflection of additional injuries, as reflected by the higher SIR in younger patients.

Although younger facial fracture patients experienced more severe injuries, the likelihood of in-hospital mortality and increased length of stay were associated with advanced patient age. The higher rate of comorbidity in older patients may explain this conundrum and be associated with higher mortality and increased length of stay. Facial fracture patients in this study did demonstrate higher rates of several comorbidities as compared to the general public, which, in addition to old age, predisposes them to higher rates of in-hospital mortality from trauma. While facial fractures are important to consider in such patients, an overall evaluation and triage of the facial fracture versus other injuries to the patient to is essential determine the best care strategy by the clinicians.

The facial fracture pattern was also affected by patient age. Zygomatic fractures were more common in older patients. In comparison, mandibular fractures were more likely in younger patients. Previous studies have indicated that mandibular fractures are associated with severe trauma [8]. Thus, mandibular fractures being more common in younger patients likely reflects the severity of injury experienced by these patients. In mandibular fractures, more force may be required to induce a fracture than other facial bones. This increased force requirement may be attributed to the mandible's greater mobility as a hinged joint, which allows for better absorption of impact and reduces the likelihood of fractures compared to other facial bones.

Younger facial fracture patients experienced injury because of non-fall-related trauma more frequently than older patients. The forces associated with various non-fall traumas are diverse, but force vectors that may target the mandible are expected in many cases. For example, head-on and lateral forces may cause injury to the mandible in a motor vehicle collision. Mandibular fractures in motorcycle, moped, and four-wheeler/ATV accidents reflect a lack of full-face helmet use. A full-face helmet has been shown to reduce the likelihood of mandibular injuries caused by collision [9]. The use of full-face helmets, especially in cases where forces are strong enough to cause severe trauma, is recommended. Further studies on the mechanism of mandibular fractures among different traumas would enable us to guide public opinion and decrease the incidence of mandibular fractures.

Female patients with facial fractures tended to be older than their male counterparts. This aligns with the average female life expectancy being longer than the average male life expectancy [10]. Moreover, there was a higher incidence of osteoporosis in females than in males [11]. Consequently, the decreased bone density associated with osteoporosis in the later stages of life makes females more vulnerable to facial fractures. This is important for clinicians to consider as older, female patients presenting after a fall should heighten clinical suspicions for facial fractures.

African Americans and other non-Caucasians were younger when experiencing facial fractures than Caucasians. This could be attributed to the fact that a lower average socioeconomic status results in a higher risk of facial trauma at a younger age. The differences between Caucasians and other non-Caucasian races require further study but may be driven by the same mechanism. Many social determinants of health are directly impacted by race. Thus, some races are likely disproportionately impacted by factors that increase the risk of facial fracture trauma at younger ages. More research needs to be done to pinpoint the specific origins of this discrepancy. Still, clinicians may show more care in assessing for facial fractures in younger, non-Caucasian patients who have suffered a trauma that could lead to a facial fracture.

This study has several limitations. The trauma registry is not solely designed for research. Furthermore, the data were drawn from a single center in a small rural community in South Carolina and may not be generalizable to larger populations or regions. Our study was retrospective in nature and thus inherently subject to reporting biases and inaccuracies that may affect the results. We recommend further research involving large multi-institutional studies or prospective observational design.

## Conclusions

Various age effects play a significant role in determining facial fracture patterns and outcomes. Younger facial fracture patients are more likely to experience more widespread and complex trauma than older patients. On the other hand, older patients are more likely to have more severe primary injuries to the face. Older facial fracture populations tend to have a higher incidence of zygomatic fractures than younger populations, who are more likely to suffer from mandibular fractures. Caucasian women were older when experiencing facial fractures, while African-American men were younger.
